# Assortment of GABAergic Plasticity in the Cortical Interneuron Melting Pot

**DOI:** 10.1155/2011/976856

**Published:** 2011-07-11

**Authors:** Pablo Méndez, Alberto Bacci

**Affiliations:** European Brain Research Institute (EBRI), Via del Fosso di Fiorano 64, 00143 Rome, Italy

## Abstract

Cortical structures of the adult mammalian brain are characterized by a spectacular diversity of inhibitory interneurons, which use GABA as neurotransmitter. GABAergic neurotransmission is fundamental for integrating and filtering incoming information and dictating postsynaptic neuronal spike timing, therefore providing a tight temporal code used by each neuron, or ensemble of neurons, to perform sophisticated computational operations. However, the heterogeneity of cortical GABAergic cells is associated to equally diverse properties governing intrinsic excitability as well as strength, dynamic range, spatial extent, anatomical localization, and molecular components of inhibitory synaptic connections that they form with pyramidal neurons. Recent studies showed that similarly to their excitatory (glutamatergic) counterparts, also inhibitory synapses can undergo activity-dependent changes in their strength. Here, some aspects related to plasticity and modulation of adult cortical and hippocampal GABAergic synaptic transmission will be reviewed, aiming at providing a fresh perspective towards the elucidation of the role played by specific cellular elements of cortical microcircuits during both physiological and pathological operations.

## 1. Introduction

The cerebral cortex (which includes the hippocampus, the entorhinal cortex, the piriform cortex, and the neocortex) is the origin of the most sophisticated cognitive functions and complex behaviors. Indeed, the constant computation of incoming sensory information is dynamically integrated to provide a coherent representation of the world, elaborate the past, predict the future, and ultimately develop a consciousness and the self. In particular, the specific activity states of intricate cortical networks often produce a wide range of rhythmic activities, believed to provide the computational substrate for different aspects of cognition and various behaviors [1, 2]. Cortical oscillations range from slow-wave activity (<1 Hz) to ultrafast oscillations (>100 Hz), with several intermediate rhythms (e.g., theta, beta gamma), each of which is considered to underlie specific cognitive aspects, such as non-REM sleep (slow-waves), sensory integration (gamma), working memory (theta), and motor planning (beta) [1]. Importantly, inhibitory neurons were proposed to play a fundamental role in the genesis of most of these rhythms [3–13] through the specialized activity of their GABAergic synapses [7–10]. In fact, it is noteworthy that malfunctioning of specific GABAergic circuits is often indicated as a leading pathophysiological mechanism (among others) of psychiatric diseases, such as schizophrenia and autism [14–18]. 

Synapses are very specialized structures responsible for the propagation of information between neurons. One of the hallmarks of synaptic transmission is its ability to be modified by certain activities or specific modulators. Modifications of synaptic strength can occur in a short- (seconds) or long-term (from hours to days) fashion. In the last decades, the plasticity of excitatory glutamatergic synapses was extensively studied as it has been proposed to be the synaptic correlate of learning and memory [19–21]. In contrast, plasticity of GABAergic synapses received less attention until recently, when it became clear that also inhibitory synapses can undergo short- and long-term plasticity [22]. However, the underlying mechanisms for GABAergic plasticity are not completely understood, given also the staggering diversity of inhibitory neurons embedded in cortical circuits and their equal heterogeneity of synaptic properties [3, 9, 23–38]. 

Here, we review some aspects of GABAergic synaptic plasticity in the context of the great disparity of GABAergic interneuron classes and the putative roles of specific changes of GABAergic synaptic strength during cortical operations. Notably, a recent review by Castillo et al. [39] covered several aspects of GABAergic synaptic plasticity, focusing on the pre- versus postsynaptic induction and expression mechanisms (see in Table 1 in [39]).

## 2. Interneuron Diversity

**Figure 1:**
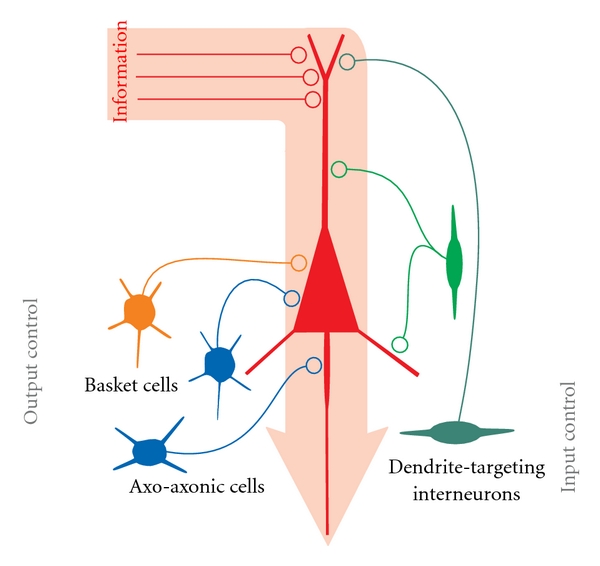
*Oversimplified scheme of the inhibitory control of cortical pyramidal neurons by several general classes of GABAergic interneurons*. Information (pink wide arrow) is transferred from excitatory glutamatergic synapses (red axon terminals) to the pyramidal neuron (red cell) dendrite. Excitation (information) travels along the dendritic tree to the soma and axon initial segment, where it could generate an action potential. Along this dendro-somatic-axonal axis, information can be differently filtered by GABAergic synapses possessing specific basic and plasticity properties. On the left-hand side, interneurons controlling the output are illustrated as different classes of basket and axo-axonic cells. Different GABAergic interneurons controlling the input into pyramidal neurons are shown on the right, as impinging the dendrite(s) at different distances from the soma. Details in the text.

In the mammalian cerebral cortex, the stereotyped interactions of multiple neuron types arranged in layers result in complex networks composed by excitatory (glutamatergic) and inhibitory (GABAergic) neurons. Although some heterogeneity of cortical excitatory neurons exists in terms of anatomy, electrophysiology, and connectivity patterns [40–46], the morphological and physiological properties of excitatory neurons are relatively homogeneous. In contrast, inhibitory neurons of cortical structures encompass a vast number of different cell types [3, 23, 34–38]. For example, in CA1 region of the hippocampus, 16 different types of interneurons have been identified so far [3]. Inhibitory neurons release GABA and are locally projecting cells, hence their “interneuron” denomination, indicating that cell body, dendrites, and axonal projections, are confined within the same anatomical area. The vast majority of interneurons show aspiny dendrites, or a relatively small spine density [47], and, unlike glutamatergic cells, they can be contacted by both glutamatergic and GABAergic synapses at the soma [48]. The classification of interneurons is based on the expression of certain calcium binding proteins and/or neuropeptides, specific electrophysiological signatures (action potential waveform and dynamic range), and functional characteristics of synapses that they form and receive, as well as specific anatomical and morphological properties [7, 25, 27, 34–38, 49, 50]. Overall, interneurons provide inhibition to neuronal networks and dictate the temporal pattern of activity of principal pyramidal and other inhibitory neurons. In this context, the rich diversity of GABAergic cells operates a division of labor during cortical activities (oversimplified in Figure 1) [11, 13], and the specific roles played by each interneuron subtype in the functional organization of cortical networks has only recently begun to be elucidated [3].

**Figure 2:**
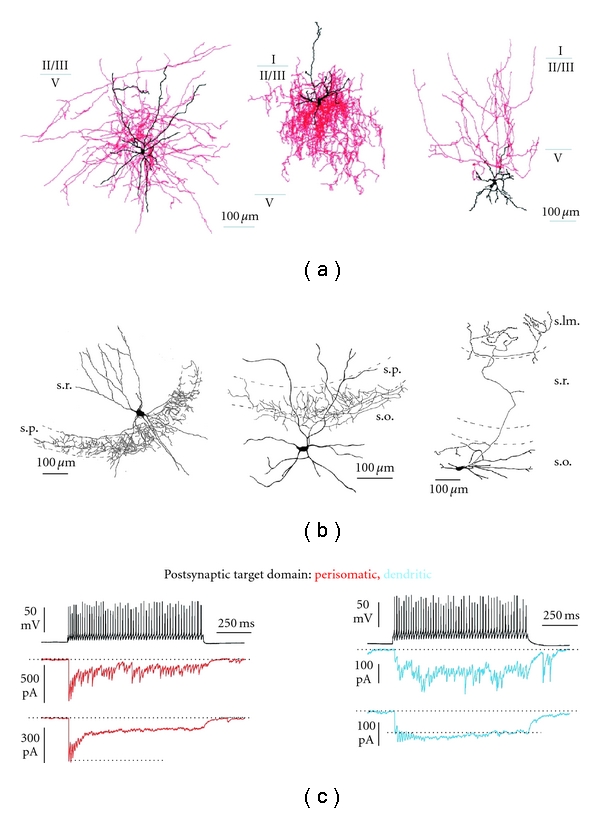
Example of diverse functional classes of inhibitory interneurons. (a) Single-neuron reconstructions of three different interneuron types of the neocortex: a basket cell (left), an axo-axonic cell (middle), and a dendrite-targeting Martinotti cell (right). Axons and somatodendritic compartments are shown in red and black respectively. Modified with permission from [51]. (b) Exampled of similar classes of GABAergic interneurons as in a, but in the hippocampus. modified with permission from [11]. (c) Different hippocampal interneuron classes show distinct properties of synaptic transmission. Examples of depressing (red traces) and facilitating (blue traces) unitary GABAergic responses originating from perisomatic and dendrite-targeting interneurons respectively. The upper red and blue traces are single-trial responses, whereas the bottom traces are averaged of multiple trials. Modified with permission from [52].

Whereas interneuron dendritic morphology is highly variable, the axonal arborization can reveal specific functional features (Figures 2(a) and 2(b)). Indeed GABAergic interneurons are specialized in targeting specific domains of excitatory principal cells, and specific patterns of axonal projection result in one of the most relevant functional classifications of interneurons. For instance, oriens to lacunosum-moleculare (OL-M) neurons in the hippocampus and their neocortical counterpart, the Martinotti cells, represent a prominent type of dendrite-targeting interneurons [28, 37, 52, 53]. Other dendrite-targeting interneurons include the neurogliaform cells [35, 54–57], the bi-stratified and tri-stratified interneurons [3, 38, 58], and ivy cells [59] in the hippocampus. All these cell types target the dendrites of pyramidal neurons (at different distances) and are thus optimally predisposed to filter synaptic glutamatergic inputs that are exclusively present on pyramidal cell dendrites (Figure 1) [41, 60]. On the other hand, basket cells (BCs, representing ~50% of all inhibitory neurons) are specialized in targeting the soma and proximal dendrites of pyramidal cells [10]. By setting the timing of action potentials of many pyramidal neurons, BCs crucially regulate the neuronal output and promote synchronous discharge of a large population of principal cells (Figure 1) [5, 6, 10]. Moving along the dendro-soma-axon line of pyramidal neurons, another type of interneuron is specialized in targeting the axonal initial segment of principal cells: the axo-axonic or chandelier cells [23, 35, 37, 61]. GABAergic synapses formed by these cells on axons of pyramidal neuron suggest a powerful role as controllers of their output (Figure 1). A clear functional distinction of the division of tasks between axo-axonic and perisomatic targeting interneurons is still unclear, as both cell types target the output region of pyramidal neurons. Interestingly, GABAergic synapses from neocortical axo-axonic cells were recently found to exert a paradoxical excitatory role, promoting action-potential generation in pyramidal neurons [62–64], although this is still matter of debate [64, 65]. 

In the hippocampus and cortex, BCs can be subdivided in two major, nonoverlapping subtypes with different physiological properties. Parvalbumin (PV) expressing basket cells can sustain high-frequency firing (hence their fast-spiking or FS denomination) and receive strong and fast glutamatergic input that relies mainly on AMPA receptors and efficiently recruits them during cortical activity [7, 25, 66, 67]. PV+ BCs are selectively surrounded by polyanionic chondroitin sulfate-rich perineuronal nets [68], which seem to play an important role in controlling ocular dominant plasticity in the neocortex [69, 70] and protect erasure of fear memories in the amygdala [71]. FS BCs release GABA very reliably due to the tight coupling between Ca^2+^ channels and Ca^2+^ sensors at their terminals [72, 73] and are extensively interconnected through chemical and electrical synapses [49, 74–78]. In particular, in the neocortex, FS BCs make a large number of synaptic contacts with themselves (autapses) [79–82] that modulate their own spike frequency and greatly contribute to improve precise spike-timing [83]. All these features allow PV+ BCs to synchronize a large population of principal cells and are thus believed to be the clockwork of cortical networks as they entrain oscillations that underlie several complex cognitive functions, including sensory integration, attention, exploratory behavior, sleep, and several forms of memory [1]. Remarkably, FS interneurons might promote network desynchronization in response to certain pattern of intense activity. This effect is mediated by massive asynchronous release of GABA from FS interneurons both at autapses and synapses with pyramidal cells resulting in reduced spike-timing precision [82]. 

In contrast, interneurons belonging to another perisomatic targeting interneuron subclass express cannabinoid receptor type 1 (CB1Rs) and the neuropeptide cholecystokinin (CCK), cannot sustain high-frequency firing, are contacted by less glutamatergic synapses, and their soma-targeted synapses tend to release GABA asynchronously and unreliably, often resulting in prolonged inhibition of target cells [30, 31]. Remarkably, GABAergic synapses formed by CCK+ BCs are negatively modulated by endocannabinoids yielding to both short- and long-term synaptic plasticity [84–86] (see below). 

Importantly, alterations of cortical inhibition were implicated in several neuropsychiatric (e.g., schizophrenia, autism, mood disorders) [14, 16–18, 87–89] and neurological (e.g., epilepsy, and Rett syndrome) diseases [90, 91]. Several lines of evidence indicate that the pathological mechanisms leading to the development of these diseases do not affect inhibitory circuits globally, but they seem to be restricted to specific interneurons types. Indeed, animal model of these diseases [92] and postmortem analysis of human tissue [93, 94] indicate a decreased number and function of PV+ BCs. In line with these anatomical results, abnormal oscillatory activity was associated to schizophrenia, autism, and epilepsy [95, 96]. Conversely, the prominent subcortical aminergic input to CCK basket cell [97, 98] has prompted the hypothesis that this particular BC subtype is the substrate of plastic changes that control mood and its disorders [10]. However, an increasing amount of evidence suggests that PV+ basket cells are indeed the target of several neuromodulators such as CCK, opioids, and serotonin [99–101] and could be affected by hormones and stress that has a facilitating role towards the development of depressive disorders [102, 103].

## 3. Plasticity of Adult GABAergic Synapses: Cellular Mechanisms

Since the discovery of activity-dependent potentiation of synaptic strength in the hippocampus [104], considerable effort has been done to elucidate the mechanisms underlying the plasticity of glutamatergic transmission as it is supposed to rule the functional and structural refinement of synaptic contacts and be the neuronal correlate of learning and memory [20]. Conversely, plasticity of GABAergic synaptic transmission has received much less attention, but an increasing effort made during the last two decades is starting to give us some cues about the mechanisms and roles of inhibitory plasticity. Today, there are examples of GABAergic plasticity in many different brain areas such as cerebellum, brain stem, deep cerebellar nuclei, VTA, thalamus, lateral superior olive, and amygdala [22]. In the cortex and hippocampus, both long- and short-term changes in GABA transmission were described [22]. 

### 3.1. Retrograde Synaptic Signaling and GABAergic Plasticity

**Figure 3:**
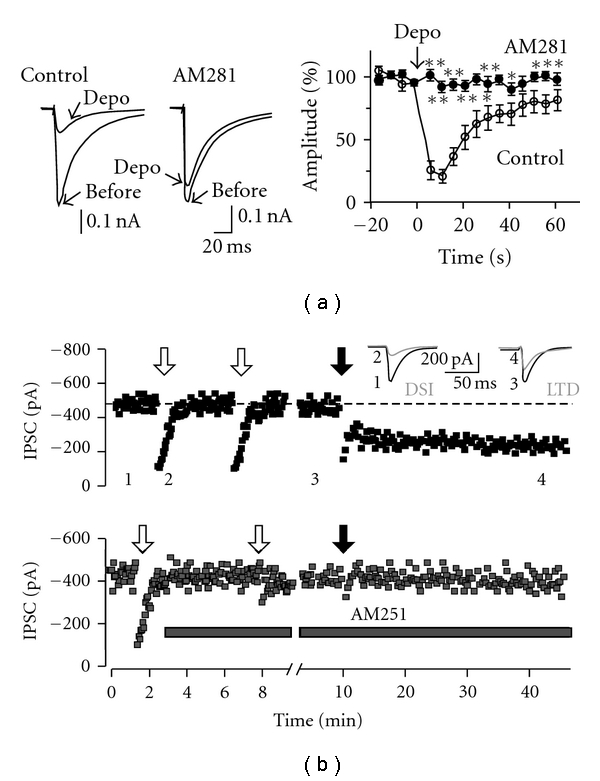
*Endocannabinoid-dependent plasticity of GABAergic synapses*. (a) In cultured hippocampal neurons, eCBs mediate a form of short-term retrograde signaling strongly reducing GABAergic responses. This can be observed by the reduction of unitary inhibitory postsynaptic currents (IPSCs) evoked after a 5 sec-long depolarization (depo) of the postsynaptic neuron. The CB1R antagonist AM281 blocked the depolarization-induced suppression of inhibition (DSI). Time course of DSI is indicated in the right panel. Modified from [105]. For details, see reference [105, 106]. (b) Time course of extracellularly evoked IPSC amplitude in the CA1 area of the hippocampus. Brief depolarizations (white arrows) of the recorded pyramidal cell induce DSI (see Figure 3), whereas high-frequency stimulation (black arrow) of afferent fibers induces LTD of GABAergic responses. Traces correspond to the time points indicated by numbers in the upper graph. Both DSI and LTD induction are blocked by the selective CB1R antagonist AM251 (gray bar, lower graph). Modified with permission from [107].

Retrograde synaptic signaling has emerged as one of the major mechanisms for GABAergic synaptic plasticity. Indeed, postsynaptic depolarization- or activity-dependent short-term suppression of presynaptic GABA release was described in the early 90s in the hippocampus and cerebellum and termed depolarization-induced suppression of inhibition (DSI) [108, 109]. In 2001, it was shown that endogenous cannabinoids (or endocannabinoids; eCBs) are the actual retrograde messengers mediating this post- to presynaptic communication (Figure 3(a)) [105, 106, 110–113]. eCBs are ubiquitous signaling molecules through the CNS. In the cortex and hippocampus, 2AG and anadamide, the two major endogenously produced cannabinoids [106, 114–116], are responsible for different forms of plasticity of GABAergic neurotransmission, including short- and long-term modification of synaptic strength and homo- and heterosynaptic forms of plasticity [85, 107, 111]. eCBs can be synthesized on demand, in response to many stimuli such as postsynaptic depolarizations, increased Ca^2+^ concentrations, action potential trains and metabotropic glutamate (mGlu), dopamine, and acetylcholine receptor activation [106]. After their synthesis, eCBs travel backwards from the postsynaptic cell—where they are produced—to presynaptic terminals and generate a short-term (seconds to minutes) and/or long-term (minutes to hours) suppression of GABA release through activation of CB1 receptors, G-protein coupled receptors, located mainly on presynaptic terminals [85, 106, 114]. Distinct stimuli set the duration of CB1R-mediated plasticity by activating different downstream signaling mechanism. Short-term postsynaptic depolarization results in short-term GABAergic transmission inhibition, (DSI, Figure 3(a)) that occurs through inhibition of voltage-dependent calcium channels by CB1Rs [106, 107]. Intense high-frequency synaptic stimulations of afferent fibers induce a long-term disinhibition of pyramidal cells in CA1 area of the hippocampus (Figure 3(b)) [86, 107, 111]. This form of long-lasting plasticity of GABAergic transmission, termed eCB-dependent long-term depression (eCB-LTD), depends on CB1R-mediated regulation of presynaptic protein kinase A (PKA) and the phosphatase calcineurin [117, 118]. These two signaling proteins control a cascade that results in long-term inhibition of the presynaptic release machinery.

**Figure 4:**
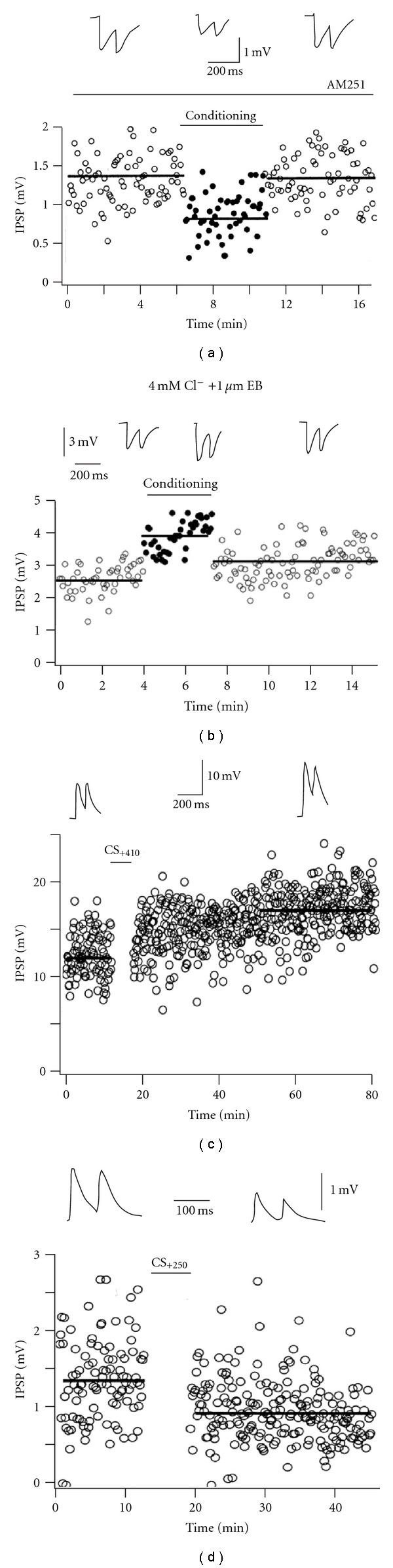
*Endocannabinoid-independent plasticity of GABAergic synapses*. (a) Brief train of action potentials (conditioning) in cortical pyramidal cells depresses unitary inhibitory postsynaptic potentials (uIPSPs) evoked by synaptically connected FS interneurons (top traces, from left to right: before, during and after conditioning). This form of short-term retrograde depression is indicated by the black dots during the conditioning paradigm (conditioned response is measured 250 ms after the conditioning stimulus), and it is not blocked by the selective CB1R antagonist AM-251, ruling out the involvement of eCB signaling. (b) Conditioning mediated depression of uIPSPs from FS interneurons (top traces, from left to right: before, during, and after conditioning) is prevented by the nonselective vesicular glutamate transporter Evans Blue (EB) suggesting a critical role for dendritically released glutamate in this form of plasticity. Modified with permission from [119]. For details see [119, 120]. (c, d) Spike timing-dependent plasticity (STDP) results in potentiation (c) and depression (d) of uIPSPs (top) elicited by FS interneurons onto cortical pyramidal cells. Long-term potentiation (LTP) of uIPSP amplitudes (top traces) is obtained when presynaptic FS cell fires 410 ms after the beginning of a brief train of action potentials (10 action potentials at 50 Hz) in the postsynaptic pyramidal cell (c). Conversely, long-term depression (LTD) of uIPSPs is observed when the presynaptic FS cell fired 250 ms after the start of an identical train (d). (c and d): Modified with permission from [121].

Another form of eCB-independent retrograde signaling has been described in cortical GABAergic synapses formed by nonaccommodating FS cells and pyramidal cells in layer 2/3 of the cortex. Zilberter showed that increase of postsynaptic pyramidal-cell Ca^2+^ concentrations induced by trains of action potentials results in a short-term decrease of GABAergic transmission between these two cell types [120]. Pair-pulse ratio analysis indicated a presynaptic locus for this phenomenon and suggested the involvement a retrograde signal. Although increases in pyramidal neuron dendritic Ca^2+^ levels are a triggering signal for the synthesis of eCBs, FS cells in L2/3 of the cortex do not express detectable CB1Rs, therefore ruling out the participation of eCBs in this form of plasticity [119]. Further investigations have shown that this form of disinhibition is likely mediated by somatodendritic release of glutamate-filled vesicles expressing the vesicular glutamate transporter vGLUT3 with consequent activation of presynaptic metabotropic glutamate receptors (Figures 4(a) and 4(b)) [119, 120].

### 3.2. Spike Timing-Dependent Plasticity of GABAergic Synapses

Spike timing-dependent plasticity (STDP) is a form of synaptic plasticity that requires both pre- and postsynaptic firing, inducing changes in synaptic strength whose polarity (potentiation or depression) depends on the temporal order of pre- and postsynaptic spiking. Glutamatergic STDP has been shown to follow precise general rules: long-term potentiation (LTP) of synaptic transmission is produced when presynaptic spiking precedes (in a millisecond time window) postsynaptic action potential, whereas LTD is induced when postsynaptic spikes precede presynaptic action potentials [122–124]. STDP of GABAergic synapses (and of glutamatergic synapses onto inhibitory cells [125]) has only recently been investigated and seems a bit more complex than glutamatergic STDP. Indeed, in the hippocampus, a symmetric dependency was found: LTP of GABAergic connections was induced when pre- and poststimuli where paired at ±20 milliseconds whereas longer intervals led to LTD [126]. Conversely, in the entorhinal cortex, GABAergic STDP follows the same temporal dependency as glutamatergic STDP [127]. Both hippocampal and entorhinal cortex spike-timing LTPs depend on postsynaptic Ca^2+^ rises induced by back-propagating action potentials and were proposed to have a postsynaptic origin [126, 127]. Interestingly, in hippocampal neurons (both cultured and in slices), it has been shown that coincident pre- and postsynaptic firing that results in LTP of GABAergic transmission produced a shift of the reversal potential for GABA-mediated (E_GABA_) responses at this particular synapse. Indeed, the coincident activity resulted in the inhibition of the Cl^−^ cotransporter KCC2 resulting in a more depolarized E_GABA_ [126]. 

Given the rich heterogeneity of GABAergic interneuron subtypes, one key question is whether plasticity of GABAergic neurotransmission follows some general rules regardless of the GABAergic cell subtype or if specific inhibitory cell subclasses are more susceptible to develop certain forms of plasticity. Remarkably, Holmgren and Zilberter demonstrated that in neocortical layer 2/3 unitary connections between FS interneurons and pyramidal neurons are substrate for long-term modification of synaptic strength induced by pairing pre- and postsynaptic action potentials [121]. Indeed, this study showed that LTP of GABAergic responses was induced when the presynaptic FS cell fires at least 400 ms after the postsynaptic pyramidal did. Interestingly, the plasticity of this particular GABAergic synapse is bidirectional and LTD was induced if presynaptic FS fires during or shortly after a train of action potentials in a pyramidal cell (Figures 4(c) and 4(d)) [121]. In contrast with the results observed in hippocampal cells, STDP of FS to pyramidal neurons did not alter the reversal potential for synaptic responses, suggesting an alternative mechanism for this form of plasticity [121]. Although the exact mechanism leading to STDP of FS to pyramidal cell GABAergic transmission is still unknown, the dependency on intact calcium signaling and unchanged pair-pulse ratio of unitary postsynaptic responses after conditioning does not favor a presynaptic origin [121]. In line with a postsynaptic expression of GABA-mediated synaptic plasticity onto neocortical pyramidal neurons, recent evidence indicated the role of postsynaptic L- and R-type Ca^2+^ channels in activity state-dependent LTD and LTP of GABAergic inhibition in layer 5 pyramidal neurons [128].

### 3.3. Other Types of Plasticity of GABAergic Synaptic Transmission

Activity-dependent plasticity of GABAergic synapses has been demonstrated in adult cortex and hippocampus. Both LTP and LTD of GABAergic transmission can be triggered by different forms of stimuli that consist mostly in high-frequency afferent stimulations [86, 129–132]. Several forms of heterosynaptic long-term changes of GABAergic responses were shown in adult hippocampus and have the activation of glutamatergic fibers as a common origin [86, 129]. Although induction is invariably postsynaptic, the expression locus can be either pre- or postsynaptic. In CA1 region of the hippocampus, glutamate released by Schaffer-collaterals activates mGluRs, triggering the synthesis of eCBs that act presynaptically to reduce GABA release (see above) [86, 107, 111]. Notably, a different study reported that glutamate induces postsynaptic Ca^2+^ increases through NMDA receptors that, in turn, activate postsynaptic calcineurin [129]. Importantly, this calcium-sensitive phosphatase has been involved in the negative regulation of GABA_A_ receptors activity resulting in a postsynaptic locus of expression for this form of GABAergic LTD [129]. As a common theme, it seems that the induction of all these forms of GABAergic plasticity requires the sustained firing of the GABAergic cell that produced GABAergic LTD. This suggests a dual role of GABAergic interneurons: promoting synaptic plasticity and conferring synapse specificity [117, 133–136]. 

Another form of activity-dependent potentiation of inhibitory synaptic transmission is mediated by astrocytic calcium signaling in the hippocampus. In synaptically coupled pairs of interneurons and pyramidal cells, a train of high-frequency action potentials in the presynaptic inhibitory cell produces an increase in the probability of GABA release that lasted for 15–20 minutes [137]. Strikingly, neighboring astrocytes were shown to be critical mediators of this effect. Indeed, interneuron firing and consequent release of GABA triggered GABA_B_-mediated calcium signaling in astrocytes adjacently located to the inhibitory neuron. Upon GABA_B_ receptor activation and through a mechanism dependent on AMPA and NMDA receptors, astrocytes induced potentiation of inhibitory transmission between interneuron and pyramidal cells [137].

Another form of GABAergic synaptic potentiation has been described in FS to stellate cells connections in layer 4 of mouse visual cortex [138]. At this synapse, paring of presynaptic FS spikes with subthreshold depolarization of postsynaptic stellate cells resulted in a significant potentiation of the GABAergic synapses that lasted for at least 30 minutes. In this study, no changes in the PPR were detected and the reversal potential of synaptic responses remained unaltered [138]. Interestingly, this form of plasticity is prevented by coupling pre- and postsynaptic spikes suggesting that STDP at neocortical FS to principal cell connections is layer dependent.

## 4. Functional Role of GABAergic Plasticity

Many examples of GABAergic synaptic plasticity come from studies focused on the development of cortical inhibitory circuits. Indeed, in the developing mouse neocortex, GABA levels are modulated by neuronal activity and sensory experience through the regulation of the *Gad1* gene [139, 140], which codes for GAD67, a glutamic acid decarboxylase that is the rate-limiting enzyme responsible for GABA synthesis [141]. In turn, modified GABA transmission increases the number of synaptic contacts, axon branching, and innervation field of single perisomatic interneurons [142, 143]. In the dentate gyrus, both pre- and postsynaptic changes occur during development of GABAergic synapses originating from PV+ BCs, including increased amplitude, decreased failure rate, and decay constant of unitary inhibitory responses [144]. These changes reflect a developmentally regulated plasticity of FS cell-mediated GABAergic transmission transforming this cellular element into the well-known precise synaptic metronome and fast signaling unit.

Despite the growing evidence in favor of GABAergic transmission as a pivotal mechanism for several functions of neuronal circuits, little is know about the actual role of activity-dependent modifications of inhibitory synapses in altering network activities that are strongly dependent on specific GABAergic circuits. In fact, functional consequences of changes in inhibitory synapse strength can vary dramatically depending on the interneurons subtype involved. Indeed, different interneuron subclasses possess different mechanisms underlying basic GABAergic transmission, such as, for example, different expression of presynaptic voltage-gated Ca^2+^ channels and/or metabotropic receptors that modulate GABA release [10, 52]. Since these differences result in specific modes of GABAergic transmission, it is likely that specific GABAergic synapses originating from specific interneuron types will generate different forms of plasticity in response to similar activity patterns. To complicate things even further, different classes of inhibitory interneurons are activated by glutamatergic synapses exhibiting peculiar properties, including short- and long-term plasticity and expression of specific ionotropic and metabotropic glutamate receptors [26–29, 33, 37, 145]. This diversity of excitatory properties onto different interneuron classes was shown to underlie differential temporal recruitment of different GABAergic cell types during cortical activities [146], therefore limiting or promoting induction of downstream GABAergic plasticity in selective cell types. Some indirect evidences for plasticity of GABAergic transmission arising from specific interneuron types were found in development when sensory activity is a critical regulator of GABAergic plasticity. For example, FS cell-mediated transmission in visual cortex was shown to develop an LTP at these inhibitory synapses in mice that were visually deprived (see above) [138]. In neocortical low-threshold spiking interneurons (including dendrite targeting Martinotti cells) similar sensory deprivation (whisker trimming) induced a change in the pattern of inhibitory transmission, with increased amplitude and decay kinetics [147]. On this line, sensory deprivation induces a decrease in the number of dendrite targeting GABAergic synapses in L4 [148] and somatic targeting interneurons [149].

The induction of plastic changes in GABAergic synapses may have different outcomes depending not only on the polarity or duration of the change, but also on the location and origin of these GABAergic synapses. In the hippocampus, stimuli that induce LTP of glutamatergic transmission also induce eCB-dependent LTD of GABAergic synapses. This form of LTD is likely restricted to interneuron types expressing CB1 receptors that include CCK-positive basket cells and Schaffer collateral-associated (SCA) interneurons [85, 150]. The plasticity of this GABAergic input has been shown to be responsible for the increased excitability of pyramidal cells after eCB signaling activation and for the EPSP-to-spike (E-S) coupling, that is, an important component of LTP of glutamatergic transmission [129].

The increased strength of GABAergic transmission between PV+ BCs and pyramidal neurons would decrease the excitation-to-inhibition ratio in the somatic compartment of principal cells and limit their time window for spike generation. Since each PV+ BC contact a large number of pyramidal neurons, the plasticity of its GABAergic connections will influence a large portion of the network, and therefore change some global properties of network activities. This applies if plasticity of GABAergic synapses results from a broad change of presynaptic neurotransmitter release, regardless of postsynaptic activity. On the other hand, combined presynaptic and single pyramidal neuron firing might induce STDP modifying a small portion of GABAergic synapses. This can happen during theta and gamma activities, when the firing of pyramidal neurons and FS cells are temporally displaced as they are locked to different components of the oscillation phase [3, 13]. Another form of fine regulation of few components of a network is represented by eCB-dependent synaptic plasticity. Indeed, eCB-mediated decrease of perisomatic inhibition arising from CCK+ interneurons will likely disinhibit and thus increase excitability of those single pyramidal cells that retrogradely delivered eCBs. This mechanism will therefore provide a self-induced fine tuning of inhibition. In addition, since these signaling molecules are produced by highly active principal cells, eCBs are ideally placed to organize cell assemblies that fire in close relation during certain behavioral states, although the role of eCB-mediated retrograde signaling onto CCK+ cells during oscillations and network activities is far from being clear [151, 152]. In this scenario, it is possible that sustained firing activities of pyramidal cells will induce an eCB-dependent overall depression of GABAergic transmission originating from CCK+ interneurons. This will likely shift the balance of perisomatic inhibition towards the fast, precise, and reliable inhibition from PV+ basket cells, which are insensitive to eCBs. Since these two types of interneurons differentially contribute to feed forward and feed back inhibition onto CA1 cells, retrograde eCB signaling has the potential of changing the integration properties of principal cells by narrowing the time window for spike generation and allowing increased temporal resolution [10, 146]. As detailed above, neocortical pyramidal cells use different mechanisms to selectively modulate specific sources of perisomatic GABAergic transmission in a retrograde fashion (eCBs in CCK+ basket cellls versus glutamate in FS interneurons). It is still unclear, however, if these two modulation mechanisms can be uncoupled, thus leading to a change in the perisomatic inhibition balance originating from PV+ and CCK+ basket cells.

Synaptic plasticity of GABAergic synapses can be target specific. It has been shown that eCB-mediated suppression of GABA transmission is present at GABAergic synapses on pyramidal neurons but not on interneurons in layer 2/3 of the mouse neocortex [153, 154]. In the hippocampus, however, both GABAergic synapses on interneurons and pyramidal cells can be modulated by retrograde eCB signaling [150]. In addition, GABAergic inputs to layer 5 pyramidal cells in the neocortex is cannabinoid-insensitive, whereas GABAergic synapses onto layer 2/3 principal cells are strongly modulated by retrograde eCB signaling [155, 156]. These observations raise the possibility that certain forms of eCB-mediated plasticity may rely on the identity and location of both pre- and postsynaptic neurons. Therefore, specific activities can differentially suppress inhibition in distinct cortical layers and specific cell types (glutamatergic versus GABAergic).

There is little (if any) direct evidence for plasticity of GABAergic transmission at distal dendritic sites, such as that provided by O-LM interneurons and Martinotti cells in the hippocampus and neocortex, respectively. Importantly, Martinotti cells mediate a prominent disynaptic dendritic inhibition triggered by high-frequency firing of pyramidal neurons [157–159]. Plasticity of these GABAergic connections will, therefore, be crucial for information filtering by these dendrite-targeting interneurons [160]. 

Interestingly, the polarity of STDP of glutamatergic synapses depends on the location of the synapses within the dendritic arbor. The same timing of pre- and postspiking gives rise to LTD at most distal synapses, but LTP at more proximal dendritic synapses [161]. It will be interesting to investigate whether interneurons targeting different compartments of principal cells, for example, dendritic versus somatic, have different plasticity rules and whether specific patterns of network activation have differential effects on inhibition arising from specific sources.

## 5. Conclusions and Future Directions

In this paper we emphasized how the great diversity of interneuron types gives rise to an even greater diversity of GABAergic transmission and plasticity. Indeed, the specific key role of each GABAergic circuit in sculpting different forms of cortical activity has only recently begun to be elucidated [3]. Since it has been shown that GABAergic synapses exhibit plasticity, it will be fundamental to reveal the governing rules of GABAergic transmission originating from different neuron subclasses.

In addition to the interneuron type-specific forms of synaptic plasticity, several open questions remain, such as, for example: (i) what are the physiological activities (single neuron and/or network activities) necessary to induce plasticity of GABAergic synapses? (ii) Is there a heterogeneity or bidirectional plasticity of GABAergic synapses in different cortical areas? (iii) What is the functional role of GABAergic transmission during different cortical activities? (iv) What other neuromodulators, in addition to endocannabinoids and glutamate, can induce activity-dependent changes of GABAergic synaptic strength? (v) Could GABAergic plasticity lead to complex Cl^−^ gradients inside a principal neuron [126], such that the direction (inhibition versus excitation) of GABA-mediated responses might, in some cases, contribute to some forms of hyperexcitability? (vi) Is plasticity of inhibitory synapses altered in pathological situations? Addressing these questions will help define the fundamental molecular, cellular, and synaptic mechanisms governing several core functions of cortical activities, therefore advancing our knowledge on the basic rules underlying complex cognitive and behavioral functions, with likely important implications for neurological and psychiatric diseases.
